# Diabetic microvascular complications and associated factors in patients with type 2 diabetes in Southern Ethiopia

**DOI:** 10.3389/fendo.2024.1342680

**Published:** 2024-07-04

**Authors:** Fasika Merid, Firdawek Getahun, Habtamu Esubalew, Tamirat Gezahegn

**Affiliations:** ^1^ Department of Public Health, Arba Minch College of Health Science, Arba Minch, Ethiopia; ^2^ Department of Public Health, College of Medicine and Health Science, Arba Minch University, Arba Minch, Ethiopia

**Keywords:** microvascular complications, prevalence, associated factors, T2DM, southern Ethiopia

## Abstract

**Background:**

Microvascular complications are long-term complications that affect small blood vessels, usually developed in diabetes, and are primary causes of end-stage renal disease, several painful neuropathies, and blindness. Thus, this study aimed to determine diabetic microvascular complications and factors associated with them among patients with type 2 diabetes.

**Methods:**

An institution-based cross-sectional study was conducted among 378 type 2 diabetes patients. The presence of at least one diabetic microvascular complications diagnosed by physicians and found on the record was considered to have microvascular complications. The data was collected by reviewing the medical records of T2DM patients who were on follow-up from January 1, 2012, to December 31, 2021. The collected data was entered into EpiData version 3.1 and analyzed by Stata version 14. Bivariate and multivariable logistic regression were used to identify statistically significant risk factors for diabetic microvascular complications at p-value < 0.05.

**Results:**

Patients with type 2 diabetes mellitus had a prevalence of diabetic microvascular complications of 26.5% (95% CI: 22.0%, 30.9%). Diabetic neuropathy was the highest (13.2%), followed by diabetic nephropathy (12.4%), and diabetic retinopathy (6.4%). Increasing age, poor glycemic control, hypertension comorbidity, anemia, positive proteinuria, a longer duration of type 2 diabetes mellitus, and hypercholesterolemia were significantly associated factors with diabetic microvascular complications.

**Conclusion:**

Diabetic microvascular complications were highly prevalent. Therefore, the study suggests that interventional strategies should be taken for poor glycemic control, hypertension comorbidity, anemia, positive proteinuria, and hypercholesterolemia to control the development of diabetic microvascular complications in patients with type 2 diabetes.

## 1 Introduction

Diabetes mellitus (DM) is a chronic metabolic disease characterized by elevated levels of blood sugar, which over time leads to serious damage to the heart, blood vessels, eyes, kidneys, and nerves (1). It is found worldwide in every population in all regions, and its prevalence is continuously increasing (2). According to the International Diabetes Federation, in 2021, the prevalence of people living with diabetes was estimated at 537 million, or 10.5% of the global adult population aged 20–79 years, and it is projected to rise to 783 million in 2045 (3). Type 2 diabetes mellitus (T2DM) is the most common and accounts for more than 95% of people with diabetes (1, 4, 5). Globally, the rising tide of physical inactivity, energy-dense diets, and obesity has resulted in an unprecedented increase in the number of patients with T2DM (6).

The development of diabetic microvascular complications is significantly impacted by the rising prevalence of diabetes and the increase in life years spent with it. In addition to this, it places a huge burden on almost every health care system, both societal and financial (7). T2DM and its complications have made a significant global contribution to the burden of death and disability (8). Microvascular complications are long-term complications that affect small blood vessels, usually developed in diabetes. These typically include diabetic nephropathy, diabetic neuropathy, and diabetic retinopathy and are primary causes of end-stage renal disease, several painful neuropathies, and blindness respectively (9, 10). It also lowers the standard of living and increases medical expenses for T2DM patients (11, 12).

Microvascular complications are present in half of patients with T2DM (8). In previous studies conducted, the prevalence of microvascular complications was 18.0%-57.5% in Asia (13–16), 34.3%-48.4% in the Middle East (17–19), 47.8% in Nigeria (20), and 19.5%-42.6% in Ethiopia (21–26). Duration of diabetes, hypertension, triglycerides, age, dyslipidemia, poor glycemic control, sex, systolic blood pressure, and positive proteinuria were commonly associated factors with the development of diabetic microvascular complications in previous studies (13, 14, 16, 17, 21, 22, 24–27). Early detection of diabetic microvascular complications in patients with T2DM is important. In Ethiopia, complications from diabetes are a leading cause of morbidity and mortality, which has a knock-on effect on the economy (28). Even if studies have been conducted, diabetic microvascular complications continue to be a public health problem, and the significantly associated factors also vary in study settings. Thus, this study aimed to determine diabetic microvascular complications and factors associated with them among patients with type 2 diabetes at Hawassa University Comprehensive Specialized Hospital, Southern Ethiopia.

## 2 Materials and methods

### 2.1 Study setting and period

The study was conducted at Hawassa University Comprehensive Specialized Hospital from January 1, 2012, to December 31, 2021. The hospital is located in Hawassa town, 278 kilometers from Addis Ababa, the capital city of Ethiopia. Hawassa University Comprehensive Specialized Hospital is the biggest hospital in southern Ethiopia, providing services like emergency, orthopedic, neonatal, ophthalmic, and medical referral clinic for more than 20 million people.

### 2.2 Study design and population

Institution based cross-sectional study was conducted. The source population was all T2DM patients who were attending the diabetic follow-up clinic at Hawassa University Comprehensive Specialized Hospital. All selected newly diagnosed T2DM patients who were enrolled from January 1, 2012, to December 31, 2021, were part of the study population.

### 2.3 Sample size determination and sampling technique

The sample size was calculated by using the formula to estimate a single population proportion with the assumptions of a 95% confidence interval Zα/2 = 1.96, proportion (P) = 37.9% from a previous study conducted on the prevalence of microvascular complications (24), margin of error (d) = 0.05, and a 10% non-response rate.


$$\text{n}=\;(\text{Zα}/2{)}^{2}\;\text{p}\;\left(1-\text{p}\right)\;/\;{\text{d}}^{2}\;=\;(1.96{)}^{2}\;0.379\left(0.621\right)\;/\;0.05^{2}\;=\;362$$

By adding 10%, the final sample size was 398.

Study participants were selected by a simple random sampling technique.

### 2.4 Study variables

Dependent variable.

Microvascular complications.

Independent variables.

Socio-demographic variables (age, sex, residence), clinical and treatment-related variables (anemia, hypertension, duration of diabetes, family history of diabetes mellitus, proteinuria, fasting blood sugar (FBS), high density lipoprotein (HDL), low density lipoprotein (LDL), total cholesterol, triglyceride, and type of anti-diabetic medication).

### 2.5 Operational definition

Microvascular complications: The presence of at least any one of the following diabetic microvascular complications: diabetic nephropathy, diabetic neuropathy, and diabetic retinopathy, diagnosed by physicians and found on the record. The absence of any diabetic microvascular complications was taken as no microvascular complications.

Hypertension defined as an average systolic blood pressure ≥140 mmHg, or diastolic blood pressure ≥ 90 mmHg, or both, or are on prescription medication for hypertension (29, 30).

### 2.6 Data collection tools and procedures

The data extraction checklist had socio-demographic, clinical, and treatment-related factors. The tool was developed after reviewing different literature. The data was collected by reviewing the medical records of T2DM patients who were on follow-up from January 1, 2012, to December 31, 2021. Three nurses collected the data, which was facilitated by one supervisor.

### 2.7 Data quality control

One day of training was given to data collectors prior to actual data collection on how to retrieve records and the objective of the study. A pretest using 5% of the sample size was conducted on the same setting, and based on the result of the pretest; an adjustment was made to the data extraction checklist. During data collection, the extracted data were checked for completeness and consistency, and corrections were made accordingly.

### 2.8 Data analysis

The collected data was entered, coded, edited, and cleaned using EpiData version 3.1 and exported to Stata version 14 for analysis. Descriptive statistics, including frequencies with percentage, mean with standard deviation, and median with interquartile range were performed. A bivariate logistic regression analysis was performed between dependent and independent variables. A variable in bivariate analysis with a p-value < 0.25 was used as a candidate for multivariable logistic regression analysis. In multivariable logistic regression analysis, a p-value < 0.05 was considered statistically significant. Model was built by backward stepwise elimination. Multicolinearity was checked using the variance inflation factor with a mean cutoff value < 5. The model adequacy was checked by using the Hosmer and Lemeshow goodness of fit test, which had a P value > 0.05.

## 3 Results

### 3.1 Study participant characteristics

A study included a total of 378 T2DM patients, with a response rate of 94.97%. The study participants mean age was 49.5 years, with a standard deviation (SD) of ± 12.7 years. Two hundred ninety (76.72%) of the respondents were urban residents. More than four out of ten (44.44%) participants have hypertension, and 148 (39.15%) had a family history of T2DM. Of the study participants, nearly one-fourth (27.78%) of their HDL was less than 40 mg/dl. More than half (57.14%) of the participants used oral hypoglycemic agents (**Table 1**).

**Table 1:** Study participant characteristics of patients with type 2 diabetes in Southern Ethiopia (n=378).

Variable	Category	Frequency	Percentage
Age (years)	Mean ± SD 49.5 ± 12.7		
Sex	Male	207	54.76
	Female	171	45.24
Residence	Urban	290	76.72
	Rural	88	23.28
FBS (mg/dl)	Median (IQR) 170.5 (85.5- 255.5)		
Hypertension	Yes	168	44.44
	No	210	55.56
Family history of T2DM	Yes	148	39.15
	No	230	60.85
Anemia	Yes	17	4.50
	No	361	95.50
Positive proteinuria	Yes	82	21.69
	No	296	78.31
Duration of T2DM	< 5 years	231	61.11
	≥ 5 years	147	38.89
Triglyceride	< 150 mg/dl	224	59.26
	≥ 150 mg/dl	154	40.74
HDL	< 40 mg/dl	105	27.78
	≥ 40 mg/dl	273	72.22
LDL	< 100 mg/dl	259	68.52
	≥ 100 mg/dl	119	31.48
Total cholesterol	< 200 mg/dl	248	65.61
	≥ 200 mg/dl	130	34.39
Treatment	Oral hypoglycemic agent	216	57.14
	Insulin	124	32.80
	Both	38	10.05

FBS, fasting blood sugar; T2DM, type 2 diabetes mellitus; HDL, high density lipoprotein; IQR, interquartile range; LDL, low density lipoprotein; SD, standard deviation.

### 3.2 Diabetic microvascular complications and associated factors

According to this study, diabetic microvascular complications in patients with T2DM were 26.5% (95% CI: 22.0%, 30.9%). Diabetic neuropathy was the highest (13.2%), followed by diabetic nephropathy (12.4%), and diabetic retinopathy (6.4%). In bivariate logistic regression analysis, age, residence, FBS, hypertension, anemia, positive proteinuria, duration of T2DM, triglyceride, HDL, LDL, and total cholesterol were factors significantly associated at a p-value of 0.25. But, after adjusting for other variables in multivariable logistic regression, age, FBS, hypertension, anemia, positive proteinuria, duration of T2DM, and total cholesterol were statistically and significantly associated factors with diabetic microvascular complications in patients with T2DM. For each one year increases in age, the odds of developing microvascular complications increase by 4% (AOR, 1.04; 95% CI (1.02-1.06)). As FBS level increases by 1 mg/dl, the odds of developing microvascular complications increase by 1% (AOR, 1.01; 95% CI (1.00-1.01)). The odds of developing microvascular complications with hypertension comorbidity were 86% higher compared to diabetic patients with no comorbid hypertension (AOR, 1.86; 95% CI (1.08- 3.19)). Type 2 diabetic patients with anemia were 3.4 times more likely to develop microvascular complications as compared to their counterparts (AOR, 3.40; 95% CI (1.11- 10.44)). The odds of developing microvascular complications among type 2 diabetic patients were 86% higher in those with positive proteinuria than in those with negative proteinuria (AOR, 1.86; 95% CI (1.00- 3.44)). Type 2 diabetic patients who had a duration of diabetes ≥ 5 years were nearly 3 times more likely to develop microvascular complications than those who had a duration of diabetes less than 5 years (AOR, 2.93; 95% CI (1.72- 5.01)). The odds of developing microvascular complications among type 2 diabetic patients with total cholesterol ≥ 200 mg/dl were nearly 2 times higher compared to patients with total cholesterol less than 200 mg/dl (AOR, 1.93; 95% CI (1.13- 3.29)) (**Table 2**).

**Table 2:** Multivariable logistic regression analysis for factors associated to diabetic microvascular complications among patients with type 2 diabetes in Southern Ethiopoia (n=378).

Variable	Microvascular complication		COR 95% CI	AOR 95% CI	P-value
	No	Yes			
**Age**			1.05 (1.03-1.07)	1.04 (1.02-1.06)	0.001
Residence					
Urban	208(55.03)	82(21.69)	1.53 (0.86- 2.73)	1.59 (0.84- 3.02)	0.154
Rural	70(18.52)	18(4.76)	1	1	
**FBS**			1.01 (1.01-1.01)	1.01 (1.00-1.01)	< 0.001
Hypertension					
Yes	105(27.78)	63(16.67)	2.81 (1.75- 4.50)	1.86 (1.08- 3.19)	0.025
No	173(45.77)	37(9.79)	1	1	
Anemia					
Yes	8(2.12)	9(2.38)	3.34 (1.25- 8.91)	3.40 (1.11- 10.44)	0.033
No	270(71.43)	91(24.07)	1	1	
Proteinuria					
Yes	54(14.29)	28(7.41)	1.61 (0.95- 2.74)	1.86 (1.00- 3.44)	0.049
No	224(59.26)	72(19.05)	1	1	
Duration of T2DM					
< 5 years	191(50.53)	40(10.58)	1	1	
≥ 5 years	87(23.02)	60(15.87)	3.29 (2.05- 5.29)	2.93 (1.72- 5.01)	<0.001
Triglyceride					
< 150 mg/dl	178(47.09)	46(12.17)	1	1	
≥ 150 mg/dl	100(26.46)	54(14.29)	2.09 (1.31- 3.32)	1.17 (0.67- 2.04)	0.586
HDL					
< 40 mg/dl	71(18.78)	34(8.99)	1.50 (0.92- 2.46)	0.94 (0.52- 1.71)	0.844
≥ 40 mg/dl	207(54.76)	66(17.46)	1	1	
LDL					
< 100 mg/dl	201(53.17)	58(15.34)	1	1	
≥ 100 mg/dl	77(20.37)	42(11.11)	1.89 (1.17- 3.04)	1.36 (0.77- 2.41)	0.294
Total cholesterol					
< 200 mg/dl	196(51.85)	52(13.76)	1	1	
≥ 200 mg/dl	82(21.69)	48(12.70)	2.21 (1.38- 3.53)	1.93 (1.13- 3.29)	0.016

1=reference.

AOR, adjusted odds ratio; CI, confidence interval; COR, crude odds ratio; FBS, fasting blood sugar; T2DM, type 2 diabetes mellitus; HDL, high density lipoprotein; LDL, low density lipoprotein.

## 4 Discussion

This study’s main goal was to determine diabetic microvascular complications and factors associated with patients with T2DM. The overall prevalence of diabetic microvascular complications was 26.5%. Multivariable logistic regression analysis revealed that age, FBS, hypertension, anemia, positive proteinuria, duration of T2DM, and total cholesterol were the factors associated with the development of diabetic microvascular complications. The study findings would have implications for clinical and management practices to have strategies targeting old age, poor glycemic control, hypertension comorbidity, being anemic, having positive proteinuria, a longer duration of T2DM, and hypercholesterolemia for diabetes care to prevent or delay the development of diabetic microvascular complications.

The study found that 26.5% of study participants had diabetic microvascular complications. The finding was in line with a study conducted in the North West, Ethiopia 26.3% (22). However, the finding was higher than the finding of studies from Northern Ethiopia 19.5%, and Chandigarh, India 18.04% (13, 21). The finding of this study was lower than the finding of the studies conducted in Jimma, Ethiopia 41.5%, Dessie, Ethiopia 37.9%, Gondar, Ethiopia 31.33%, Ayder, Ethiopia 42.6%, Jos, Nigeria 47.8%, Tabuk, Saudi Arabia 34.3%, Doha, Qatar 48.4%, South India 52.1%, Tianjin, China 34.5%, and Ningbo, China 57.5% (14–17, 19, 20, 23–26). This discrepancy might be due to differences in diagnostic methods or criteria, sample size, and healthcare systems.

In this study, increased age was found to increase the risk of diabetic microvascular complications. The finding is consistent with a study conducted in Ethiopia, Nigeria, India, China, and Ireland (13, 16, 20, 24, 26, 27). This could be due to a combination effect of insulin resistance and predominately a loss of beta cells in advanced age, which results in poor glycemic control and greater microvascular damage (31). Higher FBS levels showed a significant association with the development of diabetic microvascular complications, as has been observed in previous studies conducted in Ethiopia and Saudi Arabia (22, 26). This might be associated with the high blood sugar level for longer periods effect of cell damage mainly occurs in capillary endothelial cells of the retina, mesangial cells of the renal glomerulus, neurons, and Schwann cells of peripheral nerves, causing diabetic microvascular complications (32).

According to the findings of this study, the risk of microvascular complications was higher in T2DM patients with hypertension. The study finding is in line with the studies done in Ethiopia, Saudi Arabia, Qatar, China, and Ireland (14, 16, 17, 19, 24, 27). The possible explanation might be due to increasing intracellular hyperglycemia through upregulation of the glucose transporter 1 (33). For every 10 mm Hg of higher systolic blood pressure, the risk of diabetic microvascular complications increased by 9% (34). Therefore, blood pressure control decreases the onset and development of microvascular complications in patients with T2DM (35). The finding of this study showed that T2DM patients with anemia have an increased risk of developing diabetic microvascular complications, which might be due to tissue hypoxia and hemodynamic effects in diabetic tissues (36). Previous studies showed an association between anemia and microvascular complications in patients with T2DM (37, 38).

The present study revealed that positive proteinuria were significantly associated factor of diabetic microvascular complications. The finding is similar with the previous studies conducted in Ethiopia (21, 22). Proteinuria itself could lead to the progression of diabetic nephropathy, which is indicative of damage to the glomerular filtration barrier (39, 40). Furthermore, in this study, the risk of diabetic microvascular complications was higher in patients with a T2DM duration ≥ 5 years. The finding is in accordance with the studies conducted in Ethiopia, Nigeria, Saudi Arabia, China, and Ireland (14, 16, 17, 20, 24). Moreover, the finding that the risk of diabetic microvascular complications was higher in patients with T2DM whose total cholesterol ≥ 200 mg/dl is consistent with previous studies done in Saudi Arabia and China (14, 17). The explanation may be that dyslipidemia may cause or exacerbate diabetic microvascular complications through alterations in the coagulation-fibrinolytic system, changes in membrane permeability, damage to endothelial cells, and increased atherosclerosis. Diabetes patients with hypercholesterolemia experienced a faster decline in their glomerular filtration rate (41). Mean total cholesterol levels for diabetics with microvascular complications were significantly higher (42). Thus, appropriate measures should be undertaken with regular monitoring of total cholesterol levels.

### 4.1 Strength and limitation

The limitation of this study is that the findings may not be generalizable to the total population because of the institution-based nature of the study, and some variables like socioeconomic status, alcohol use, and body mass index were incomplete in the patient’s medical records. As the study was cross-sectional, the cause-and-effect relationship between the associated factors and diabetic microvascular complications was impossible to determine. The strength of this study was that longer-term data were used.

## 5 Conclusion

According to this study, diabetic microvascular complications were highly prevalent. Increasing age, poor glycemic control, hypertension comorbidity, anemia, positive proteinuria, longer duration of T2DM, and hypercholesterolemia were factors independently associated with diabetic microvascular complications in patients with T2DM. Therefore, microvascular complications need to be identified early, and interventional strategies should be taken to control the modifiable risk factors poor glycemic control, hypertension comorbidity, anemia, positive proteinuria, and hypercholesterolemia associated with the development of diabetic microvascular complications in patients with T2DM.

## Data availability statement

The original contributions presented in the study are included in the article/Supplementary Material. Further inquiries can be directed to the corresponding author.

## Ethics statement

The studies involving humans were approved by Institutional Review Board of Arab Minch University, College of Medicine and Health Sciences. The studies were conducted in accordance with the local legislation and institutional requirements. Written informed consent for participation was not required from the participants or the participants’ legal guardians/next of kin in accordance with the national legislation and institutional requirements.

## Author contributions

FM: Conceptualization, Data curation, Formal analysis, Funding acquisition, Investigation, Methodology, Resources, Software, Visualization, Writing – original draft, Writing – review & editing. FG: Methodology, Supervision, Validation, Writing – review & editing. HE: Methodology, Supervision, Validation, Writing – review & editing. TG: Formal analysis, Methodology, Writing – review & editing.
